# Strengthening Integrative Microbiome Research Through Regional Leadership

**DOI:** 10.3390/ijerph22091322

**Published:** 2025-08-26

**Authors:** Filipa Godoy-Vitorino

**Affiliations:** Department of Microbiology and Medical Zoology, University of Puerto Rico, Medical Sciences Campus, San Juan, PR 00936, USA; filipa.godoy@upr.edu

**Keywords:** microbiome, training, the Puerto Rico Center for Microbiome Sciences

## Abstract

Microbiome science has revolutionized modern biology, shifting the focus from pathogens to the essential roles of beneficial microbes in health, metabolism, and ecosystems. Advances in genomic technologies like metagenomics have rapidly expanded our understanding of microbial diversity and function. Despite this progress, global microbiome research remains concentrated in high-resource regions, limiting diverse perspectives and opportunities in places like the Caribbean. This communication discusses the establishment of the first Center for Microbiome Sciences in Puerto Rico, which addresses this gap by providing local researchers with access to advanced tools, training, and infrastructure through broader collaboration. Novelty, services, and ideas on the integration of activities among local centers for the scientific improvement of the region are addressed. Additionally, how the center is poised to contribute to improving public and environmental health is also highlighted.

## 1. Introduction

The rapid growth of microbiome research has fundamentally reshaped modern biology, significantly influencing our understanding of health and disease. Microbiologists have progressively moved from studying isolated pathogenic bacteria toward exploring entire microbiomes, with a particular emphasis on beneficial microbes, their roles, and their interactions within hosts. The accessibility and affordability of sequencing technologies have significantly accelerated these advancements.

It is increasingly recognized that animals, including humans, host coevolving microbial communities, known collectively as microbiomes [1]. These microbiomes include diverse organisms such as bacteria, archaea, fungi, and viruses, which colonize epithelial surfaces and bodily cavities [2]. Despite bacteria being among the earliest forms of life on earth and extensively studied, their precise functions and impacts on health and disease remain inadequately understood.

Numerous studies have shown that beneficial microbes play a critical role in maintaining the health of nearly all organisms, including humans. Microbial cells are at least as abundant as human cells, and microbial genes outnumber human genes within the combined entity known as the holobiont [3]. These microbiomes orchestrate crucial processes like energy regulation, health maintenance, and nutrient cycling, which are essential for ecosystem stability. Advancements in metagenomics have greatly enhanced the understanding of microbial diversity, significantly expanding our knowledge beyond traditional gene-centric methods.

However, microbiome research and training is primarily conducted in research-intensive areas of the world, located largely in economically privileged regions such as North America and Europe [4]. The underrepresentation of microbiomes from other regions restricts the global diversity of scientific perspectives. The establishment of a regional center like the Centers of Biomedical Research Excellence (COBRE) Puerto Rico Center for Microbiome Sciences (NIGMS #1P20GM156713-01), addresses this disparity by expanding studies using microbiome diversity perspectives and equipping local researchers with advanced analytical tools and specialized training. This initiative promotes greater participation and enhances the capabilities of the biomedical workforce in the region, thereby fostering broader contributions to the microbiome field. The center represents a strategic initiative designed to bolster microbiome research and enhance scientific training and capacity. It plays a crucial role in advancing scientific research and fostering a vibrant community of competitive researchers.

## 2. The Microbiome as a Critical Interface Between Human and Environmental Health

Microbial–host interactions occur both locally and systemically, shaping key biological processes such as immune regulation, developmental signaling, energy metabolism, and the maintenance of epithelial integrity [5]. These topics encompass most of biology and are crucial for providing a focus for training and research. Maintaining health depends on the compartmentalization of microbiomes within specific body niches. Extensive research has characterized the bacterial communities across various human body sites, with the gut microbiome being the most intensively studied. Landmark initiatives, such as the Human Microbiome Project (HMP) [6] and MetaHIT [7], pioneered the first comprehensive gene catalogs of the human microbiota. These projects employed deep 16S rRNA gene sequencing on thousands of samples collected from men and women across body sites. Data showed that each body site hosts a highly specialized microbial community tailored to its unique environment [6,8]. Despite this niche specialization, many essential biological functions—such as nutrient synthesis and immune modulation—are conserved across body sites. Although many core microbial functions are conserved, individual variation in microbial composition is well documented and often correlates with ethnic and racial backgrounds. While studies span a wide range of body sites and global environments, most research remains concentrated in wealthier regions—surprisingly, where microbial diversity is lower. In contrast, the highest microbial diversity exists in less industrialized areas, yet these regions remain vastly underrepresented in global microbiome datasets [4,9]. Location plays a critical role in shaping the human microbiome because microbial communities adapt to the unique environmental, dietary, cultural, and genetic contexts of local populations. These adaptations influence how microbes interact with the host and, consequently, impact health outcomes. For instance, a study demonstrated that microbiome species can vary in function and health effects depending on geographic origin [10]. This study showed that disrupted development of the gut microbiota contributes to childhood malnutrition. Crucially, local strains isolated from Bangladeshi infants demonstrated greater fitness than U.S.-derived strains in gnotobiotic mouse models when tested on culturally relevant diets. This suggests that microbiota-based therapies developed in one region may have limited efficacy when applied elsewhere, reinforcing the need for regional research leadership and locally derived microbial interventions [10]. Recognizing how microbiomes co-evolve with their host populations is essential to designing effective, culturally appropriate solutions to global health challenges like malnutrition. This underscores the importance of including diverse populations in microbiome research to ensure that interventions and therapeutic strategies are effective and equitable across global communities. It is encouraging to note that microbiome datasets from historically underrepresented regions are growing—for example, Maghini et al. (2025, Nature) reported 1800 newly sequenced metagenomes from Africa, significantly enriching global microbial diversity and representation [11]. In Puerto Rico and the Caribbean there are mostly diverse catalogues of bacterial communities sequenced using 16S rRNA genes in studies directed by local researchers. Unfortunately, there are few publicly available metagenomes, except for few soil [12] and coprolite shotgun metagenomes [13,14]. This exemplifies the need for a local Microbiome center to accelerate and expand local research on microbial diversity.

Preserving microbial diversity is essential for the stability and long-term sustainability of global ecosystems and genetic resources, including those within the human body. While outdoor environments harbor significantly greater microbial diversity due to interactions with plants, animals, soil, and air, built environments (BEs) such as homes, schools, and hospitals are also dynamic microbial ecosystems shaped by human activity and environmental exposure [15]. Advances in high-throughput sequencing have deepened our understanding of microbial communities in these indoor spaces, where people play a key role in introducing and resuspending microorganisms through daily activities and contact with surfaces.

Understanding the interplay between human microbiota, environmental exposures, public health, and lifestyle is critical for advancing our knowledge of health and disease. To support this integrated vision, microbiome centers—such as the COBRE Puerto Rico Center for Microbiome Sciences—are vital. These centers are uniquely positioned to lead efforts in microbial inventorying, interdisciplinary training, and the development of regionally relevant research priorities. This mission aligns closely with the One Health framework, which emphasizes the interconnectedness of human, animal, and environmental health [16]. Microbiota serves as a biological link across these domains, reinforcing the need for collaborative, cross-sectoral approaches.

In parallel, global efforts like the Microbiota Vault Initiative [17] seek to preserve microbial diversity by archiving human, animal, and environmental microbiomes. Guided by ethical governance and equitable collaboration, the Vault provides an essential platform for safeguarding these resources for future scientific and therapeutic use. Together, such initiatives strengthen local and global infrastructure for microbiome science and ensure the long-term conservation of microbial biodiversity [17]. The Vault provides access to specialists in international regulations and ethical standards, including the Nagoya Protocol and national permitting systems that govern the transboundary movement of biological samples. This ensures fair, legal, and equitable use of genetic resources, particularly in collaborative or cross-border microbiome studies. Access to the Vault as a source of advice will also be important for expanding beyond local research and for depositing collections and redundancy in studies from Puerto Rico and other biodiverse areas. Much of the world’s microbial biodiversity—and the mechanisms by which it supports natural ecosystems and health—remains unexplored. The idea of preserving Earth’s microbial heritage mirrors the role of seed banks in conserving plant genetic diversity, ensuring these vital microbial resources are safeguarded for future discovery and use. Nonetheless, training future scientists on the importance of correctly analyzing and understanding microbiome diversity is essential; thus, regional centers that foster regional scientific development are essential to advancing science on the frontier of human and environmental health.

## 3. Role of a Microbiome Center to Foster Regional Scientific Development

Despite the Caribbean’s rich biodiversity and unique public health challenges, microbiome research from the region remains severely underrepresented in the global scientific literature [4]. Only a small fraction of studies in microbial ecology and host–microbiome interactions originate from Caribbean institutions, mostly the University of Puerto Rico. Limited access to sequencing technologies, a shortage of bioinformatics training, and insufficient funding for data analysis have severely constrained local capacity. As a result, much of the microbiome work conducted in the region remains unpublished or led by external teams with minimal local engagement—a pattern often described as “helicopter science.” Puerto Rico stands out as a leading contributor within the region, but even there, resources remain limited. These disparities underscore the urgent need for a dedicated microbiome research center in the Caribbean—one that builds infrastructure, fosters regional expertise, and ensures that local scientists are not only participants but leaders in microbiome discovery. Importantly, such a center would also address the misconception that the Caribbean is adequately represented under broader Latin American research initiatives, recognizing its distinct cultural, ecological, and health-related contexts. Emerging microbiome research from Puerto Rico is beginning to yield impactful findings that demonstrate both the scientific promise and the societal relevance of locally driven research, which will only be expanded by the PR-CMS. Recent studies provide compelling evidence that regional microbiome investigations can reveal unique microbial signatures with biomedical and ecological significance. For example, the most significant microbiota study in women in the region has already demonstrated a nonoptimal cervicovaginal microbiota dominated by *Lactobacillus iners* and vaginosis-associated bacteria among Puerto Rican women, irrespective of HPV status, suggesting microbiome-linked vulnerabilities to cervical lesions that are underexplored in global datasets, including fungi and metabolites that are be linked to dysbiosis [18,19,20,21,22]. Using animal models, Díaz-Rivera et al. (2024) identified specific bacterial genera in the oral microbiota that correlate with responses to PD-1 immunotherapy in a murine model of HPV+ oropharyngeal cancer, pointing to novel biomarkers for treatment responsiveness [23]. Similarly, in marine ecology, microbiome shifts in the endangered sea urchin *Diadema antillarum* were linked to a 2022 Caribbean die-off, possibly due to human-associated contamination, highlighting the intersection between environmental microbiology and conservation science [24]. Furthermore, insights into seasonal and life-stage changes in the gut microbiota of *Tripneustes ventricosus* have provided new insights into echinoderm biology and the effects of environmental variability [25,26,27]. Following Hurricane Maria in Puerto Rico, researchers examined the nasal mycobiome of infants exposed to post-hurricane indoor environments. Infants exposed in utero to Hurricane Maria were more likely to have nasal microbiomes dominated by *Staphylococcus* and *Streptococcus*, with significantly greater microbial richness and diversity than non-exposed infants. These findings underscore the profound influence of prenatal exposure to extreme weather on early microbial development. The enrichment of environmental bacteria in the nasal passages may have long-term implications for respiratory health, emphasizing the urgent need for research into how climate-related disasters affect microbiome-related disease risk from the earliest stages of life [28]. More recently, researchers found significant shifts in fungal communities, with higher abundances of potentially harmful fungi like *Alternaria* during the first-year post-storm. These changes may increase early-life risks for asthma and respiratory disease [29]. Also, studies on the oral microbiome, have highlighted the critical role of both bacterial and fungal imbalances in the development and progression of periodontal disease among high-risk Hispanic populations in Puerto Rico [30,31,32]. Local research revealed that oral dysbiosis is influenced not only by disease severity but also by lifestyle factors such as smoking, alcohol use, and oral sex practices [32]. They also demonstrated that environmental fungal spores, including those from outdoor air, can interact with and potentially disrupt the oral microbiome [32]. This underscores a previously unrecognized link between environmental exposures and oral health. Together, these results have important public health implications, suggesting that the oral microbiome—shaped by both internal and external factors—could serve as a valuable biomarker for disease risk and guide future prevention and intervention efforts. These are examples of some of the results that underscore the value of empowering local researchers through microbiome capacity building, enabling independent project leadership, and ensuring that Caribbean-specific health and environmental priorities are addressed. Collectively, these results not only contribute to the global microbiome literature but also emphasize the urgent need to support local infrastructure that can amplify regional voices and knowledge systems.

The establishment of the COBRE Puerto Rico Center for Microbiome Sciences (PR-CMS) represents a significant step toward advancing and expanding scientific development by addressing key needs in microbiome research. The Center will play a pivotal role in nurturing junior investigators, guiding them toward becoming competitive, independent researchers and developing independent microbiome projects. The Center will support innovative projects with broad biomedical relevance by selecting meritorious junior faculty for dedicated mentorship and providing resources for their development. A structured mentorship program that integrates guidance from program leaders, project mentors, and advisory committees ensures comprehensive professional growth. The infrastructure basically composes three areas, an administrative core, where most training and management activities derive from; a research support core, where microbiome analyses are at its heart, providing novel services for the community; and a faculty development core, offering unique opportunities for the training of faculty into more competitive researchers. This core will help faculty become better mentors, become more productive researchers in terms of lab and time management skills, grantsmanship, and navigate institutional processes. The COBRE will facilitate interdisciplinary collaborations that are essential for advancing microbiome research at both the fundamental and translational levels. It will organize faculty career enhancement activities, including laboratory management, grant writing, and manuscript preparation, while promoting a collaborative culture through seminars, conferences, and meetings. These initiatives enhance the professional skills of researchers at all levels, expanding their research capabilities and fostering collaborations in the U.S. and globally.

The dedicated Microbiome Research Core will be its primary staple, by providing the state-of-the-art instrumentation and technical expertise essential for advanced microbiome research. Most microbiome research published in Puerto Rico has depended on a single lab’s short-term data analyses support (Godoy lab), with most of the support being offered on a voluntary and collaborative basis and involving students. Additional support is often given to predominantly undergraduate institution (PUI) projects, mostly supported by PR-INBRE, whose mission is to promote biomedical research infrastructure in Puerto Rico, advancing research resources and opportunities for PUIs. An exclusive microbiome research core will be transformative for the region, as it will support meritorious scientists and students, facilitating complex genomic analyses and bioinformatics support, as well as support the development of SOPs and biosafety protocols. Additionally, workshops will be available to train students and faculty on the use of bioinformatic pipelines and best practices, including partnerships in training opportunities with other microbiome centers. Through specialized services, this research core will expand technical competencies and increase the publication output in microbiome research in Puerto Rico, positioning local researchers at the forefront of microbiome science. Overall, this center will be unique in its capacity to foster a collaborative and innovative research environment, equipping local scientists with the essential tools, resources, and expertise to significantly enhance scientific development and drive meaningful advancements in microbiome sciences.

As previously highlighted, microbiome centers significantly enhance the regional research capacity by functioning as interdisciplinary hubs [33,34]. These centers facilitate microbiome research through essential collaborations, supporting novel projects and providing critical laboratory and analytical services. Microbiome centers cultivate expertise across various career stages by serving diverse stakeholders—from faculty and graduate students to broader community members. Particularly, they bridge a crucial gap by equipping mid-career researchers with the new skills and methodologies needed to integrate microbiome studies into their existing research portfolios, effectively broadening local scientific expertise, which is the vision for the Puerto Rico Center for Microbiome Sciences. Additionally, by consolidating resources and standardizing research methodologies, centers can help overcome institutional silos, fostering a more integrated scientific community. Thus, microbiome centers serve as catalysts for developing a robust scientific ecosystem, empowering regions with diverse and highly skilled researchers capable of tackling complex health and ecological challenges collaboratively and innovatively.

## 4. Strengthening Infrastructure and Research Capabilities in Microbiome Research

This novel project is transformative at all levels, but is most important due to its unique offering of centralized support for the analysis and integration of microbial community data. This includes the characterization of microbiomes via amplicon sequencing (e.g., 16S, ITS, and 18S rRNA) and the construction of high-quality metagenomes from complex, host-associated, or environmental samples. These services will be coupled with expertise in meta-omics data integration, supporting a wide range of biomedical and ecological research applications. It will also be open to industry and medical doctors for microbiome services and reports of biodiversity with expert analyses. Designed to support both clinical and environmental studies, it will offer services to a wide range of researchers, from academic scientists to healthcare professionals and industry partners. As the first dedicated microbiome-focused core of its kind in Puerto Rico, the facility establishes a much-needed hub for microbiome science. Its primary mission is to enable innovative, interdisciplinary research that addresses questions regarding microbial diversity and function across health, agriculture, and environmental sciences. It will help design, develop, and publish innovative work in microbiome discovery. In doing so, it will complement and amplify the capabilities of existing institutional cores, creating a more integrated and efficient research ecosystem. In detail, it will have two main goals: (1) To provide a metagenomics sample preparation area that will feature automated systems for nucleic acid extraction and library preparation, enabling high-throughput sequencing of diverse biological samples—including soil, water, plant, animal, and human sources. It will support biosafety planning, study design, and downstream molecular analysis with efficient, high-quality workflows. (2) To become the first data analysis bioinformatics center that provides access to advanced computing resources and expert bioinformatics support in microbiome research, assisting researchers in processing, analyzing, and visualizing microbiome data. Bioinformaticians and microbiome scientists will support workflow design, data standardization, and reproducibility, with user guides and SOPs to ensure accessibility for all experience levels. This integrated facility will be designed to serve the PR-CMS investigators and function as a shared resource that benefits researchers across the island and links with national and international partners.

Importantly, the PR-CMS Core will also become part of the U.S. Microbiome Centers of Excellence Consortium (US-MCC) [34], positioning Puerto Rico within a broader network of collaborative microbiome institutions. Being a local center in Puerto Rico, it will also contribute to a wider regional training resource in Latin America, with collaborations with microbial ecology ambassador programs like that of the International Society for Microbial Ecology (ISME) and Applied Microbiology International (AMI). Some collaborations have already occurred, for example, between Puerto Rico and Argentina or Costa Rica [35,36], and are expected to enhance. The core and training services will be available to any regional researcher, but funds from the center will exclusively serve researchers in Puerto Rico.

This national integration will enable PR-CMS to access and share protocols, collaborate on multi-site projects, and participate in data-sharing and workforce development initiatives at scale. Through these relationships, the PR-CMS will facilitate tailored training programs and short courses to develop the skills needed by early-career investigators and student researchers, further strengthening Puerto Rico’s scientific workforce. Together, this coordinated infrastructure will expand access to critical tools and expertise, foster interdisciplinary collaboration, and create lasting capacity for excellence in microbiome science—serving Puerto Rico, the region, and the global scientific community.

## 5. Maximizing Multiple Center Grants Can Lead to Real Competitive Change

Puerto Rico has a vibrant scientific ecosystem. It benefits from an active and collaborative scientific environment that is supported by multiple NIH and NSF programs and the Puerto Rico Science, Technology and Research Trust a nonprofit organization sustaining and advancing R&D in Puerto Rico. Now, the PR-CMS, focusing on advancing microbiome-driven projects, is expected to be integrated into this environment, working synergistically with other research centers to strengthen biomedical innovation across the island. The PR-CMS Microbiome Research Core will align closely with existing NIH-supported infrastructure across Puerto Rico, including the Data Science Core (DSC), the PR-INBRE Genomics and Metabolomics Cores, the High-Performance Computing Facility (HPCF), and the Sequencing and Genotyping Facility (SGF) (P20GM103475-22). These already established cores, with sequencers, servers, and bioinformaticians, will contribute in turn to this new COBRE center. These facilities, located within the UPR system, already serve as a foundational platform for molecular and computational research. The PR-CMS Core will enhance its capacity by contributing specialized personnel, technical expertise, and complementary resources to support advanced microbiome studies and will leverage shared infrastructure for training, data analysis, and joint programming. Further integration with NIH Centers of Biomedical Research Excellence (COBRE) programs will expand scientific scope. The COBRE Phase III Center for Neuroplasticity (5P30GM149367-02), based at UPR-Rio Piedras, presents natural opportunities for interdisciplinary collaboration on gut–brain–microbiome interactions, behavior, and neurological disorders. The COBRE Center for the Promotion of Cancer Health Equity (CePCHE) (1P20GM148324-01) aligns with PR-CMS in exploring microbiome-related cancer disparities affecting Hispanic populations. Through shared efforts in basic, translational, and clinical research, these centers will collectively enhance UPR’s capacity in cancer and neuroscience. In addition, the Center for Collaborative Research in Health Disparities/RCMI program (5U54MD007600-38) at UPR supports most translational studies in health disparities affecting the Puerto Rican population and offers strong potential for collaboration in extending microbiome science to underserved communities. For studies on the human microbiome, another clear partner is *The Alliance*, which is also funded by the National Institute of General Medical Sciences (U54GM133807). This center is key in Puerto Rico for supporting clinical research and intersects three institutions across the island. Support and training in human microbiome research can also be provided by the PR-CMS to benefit researchers under NIH funding. The center will also intersect with broader environmental and sustainability efforts through CARIB-CARES (1P20CA294096-01), which links climate change, cancer, and health disparities. Environmental microbiomes and agricultural resilience are natural points of integration. Lastly, the NSF E-RISE RII project (#2435987) will offer shared opportunities in omics, AI, and computational biology. Joint training programs, co-mentoring, and collaborative research can potentially enhance the reach and impact of PR-CMS across disciplines. Together, these partnerships will form a highly synergistic research environment. The PR-CMS will serve as a catalyst, strengthening institutional capabilities, supporting faculty development and their capacity to compete, be more productive, and publish their data, expanding student training pipelines and advancing Puerto Rico’s leadership in microbiome science and global collaboration.

## 6. Implications for Health Promotion, Environmental Research, and Public Health Outcomes

The establishment of the Puerto Rico Center for Microbiome Sciences (PR-CMS) represents a transformative initiative with direct implications for health promotion, environmental research, and public well-being. As awareness of the human microbiome is emerging as a powerful catalyst for healthier living and more ecologically attuned mindsets, this center will strengthen this, as not only research but education will be supported. As highlighted in recent research [37], individuals who learn about the role of their microbiome in physical and mental health often express a greater willingness to adopt lifestyle changes such as improved diet, increased physical activity, and reduced reliance on unnecessary medications. These behavioral shifts support personal health and reduce environmental burdens linked to overconsumption and pharmaceutical waste. Moreover, recognizing the human body as a “holobiont”—a superorganism composed of human and microbial cells—encourages a broader ecological perspective, one in which microorganisms are seen not as enemies but as essential partners in health and balance. This reframing of our relationship with microbes fosters a sense of stewardship toward the body and environment alike, reinforcing the interconnectedness between personal well-being and planetary health. Educational campaigns and workshops from the center will strengthen this and impact society at large. The center is poised to uncover microbial mechanisms that influence chronic diseases, infectious outbreaks, and ecological imbalances by enabling high-resolution studies of human, animal, and environmental microbiomes. These discoveries will inform the development of targeted therapeutics, diagnostics, and public health interventions tailored to the unique needs of underserved Caribbean populations. Local researchers are expected to generate novel insights to improve health outcomes across diverse ecosystems and communities through access to state-of-the-art sequencing, metagenomics, and bioinformatics infrastructure. The center is also expected to address global challenges such as antimicrobial resistance and climate-linked health disparities by enabling interdisciplinary research on microbial functions, including in the environment, such as in soil, water, and air systems. As a hub for publishing innovative research, PR-CMS will advance evidence-based strategies that support ecosystem resilience and human health.

## 7. Conclusions

Microbial–host interactions are fundamental to health, influencing immunity, development, and metabolism, with niche-specific microbiomes—especially in the gut—providing essential yet individualized functions shaped by environment, lifestyle, and genetics. The integration of microbiome science into One Health underscores the interconnectedness of human, animal, and ecosystem health, highlighting the need for regional microbiome research centers.

The launch of the Puerto Rico Center for Microbiome Sciences (PR-CMS) marks a significant step in positioning Puerto Rico as a leader in microbiome research. As the first center of its kind in the Caribbean and a member of the U.S. Microbiome Centers of Excellence Consortium, PR-CMS will support world-class science while expanding global partnerships. With microbiome science advancing rapidly, this new Center will address critical health and environmental questions. Its research will be grounded in Puerto Rico’s public health priorities and unique ecosystems, offering global relevance and local impact. PR-CMS will combine advanced bioinformatics capabilities with local, national, and international collaboration through joint symposia and training programs.

This initiative strengthens Puerto Rico’s research infrastructure while providing a platform for inclusive discovery and innovation. Therefore, Puerto Rico is not just participating in the microbiome revolution but helping lead it.

## References

[1] Godoy-Vitorino F. (2019). Human microbial ecology and the rising new medicine. Ann. Transl. Med..

[2] Dominguez-Bello M.G., Godoy-Vitorino F., Knight R., Blaser M.J. (2019). Role of the microbiome in human development. Gut.

[3] Rosenberg E., Zilber-Rosenberg I. (2014). Microbiotas Are Transmitted Between Holobiont Generations. The Hologenome Concept: Human, Animal and Plant Microbiota.

[4] Godoy-Vitorino F. (2025). Solutions to expand microbiome sciences in the Caribbean Region: An insider’s perspective. Trends Microbiol..

[5] Chung H., Pamp S.J., Hill J.A., Surana N.K., Edelman S.M., Troy E.B., Reading N.C., Villablanca E.J., Wang S., Mora J.R. (2012). Gut immune maturation depends on colonization with a host-specific microbiota. Cell.

[6] Costello E.K., Lauber C.L., Hamady M., Fierer N., Gordon J.I., Knight R. (2009). Bacterial Community Variation in Human Body Habitats Across Space and Time. Science.

[7] Li J., Jia H., Cai X., Zhong H., Feng Q., Sunagawa S., Arumugam M., Kultima J.R., Prifti E., Nielsen T. (2014). An integrated catalog of reference genes in the human gut microbiome. Nat. Biotechnol..

[8] Lloyd-Price J., Mahurkar A., Rahnavard G., Crabtree J., Orvis J., Hall A.B., Brady A., Creasy H.H., McCracken C., Giglio M.G. (2017). Strains, functions and dynamics in the expanded Human Microbiome Project. Nature.

[9] Abdill R.J., Adamowicz E.M., Blekhman R. (2022). Public human microbiome data are dominated by highly developed countries. PLoS Biol.

[10] Barratt M.J., Nuzhat S., Ahsan K., Frese S.A., Arzamasov A.A., Sarker S.A., Islam M.M., Palit P., Islam M.R., Hibberd M.C. (2022). Bifidobacterium infantis treatment promotes weight gain in bangladeshi infants with severe acute malnutrition. Sci. Transl. Med..

[11] Maghini D.G., Oduaran O.H., Olubayo L.A.I., Cook J.A., Smyth N., Mathema T., Belger C.W., Agongo G., Boua P.R., Choma S.S.R. (2025). Expanding the human gut microbiome atlas of africa. Nature.

[12] Becraft E.D., Woyke T., Jarett J., Ivanova N., Godoy-Vitorino F., Poulton N., Brown J.M., Brown J., Lau M.C.Y., Onstott T. (2017). Rokubacteria: Genomic giants among the uncultured bacterial phyla. Front. Microbiol..

[13] Reynoso-Garcia J., Santiago-Rodriguez T.M., Narganes-Storde Y., Cano R.J., Toranzos G.A. (2023). Edible flora in pre-columbian caribbean coprolites: Expected and unexpected data. PLoS ONE.

[14] Cano R.J., Rivera-Perez J., Toranzos G.A., Santiago-Rodriguez T.M., Narganes-Storde Y.M., Chanlatte-Baik L., García-Roldán E., Bunkley-Williams L., Massey S.E. (2014). Paleomicrobiology: Revealing fecal microbiomes of ancient indigenous cultures. PloS ONE.

[15] Prussin A.J., Marr L.C. (2015). Sources of airborne microorganisms in the built environment. Microbiome.

[16] Pitt S.J., Gunn A. (2024). The One Health Concept. Br. J. Biomed. Sci..

[17] Dominguez-Bello M.G., Steiger D., Fankhauser M., Egli A., Vonaesch P., Bokulich N.A., Lavrinienko A., Hoffmann C., Zimmermann P., Muhummed A. (2025). The microbiota vault initiative: Safeguarding Earth’s microbial heritage for future generations. Nat. Commun..

[18] Vargas-Robles D., Romaguera J., Alvarado-Velez I., Tosado-Rodríguez E., Dominicci-Maura A., Sanchez M., Wiggin K.J., Martinez-Ferrer M., Gilbert J.A., Forney L.J. (2023). The cervical microbiota of Hispanics living in Puerto Rico is nonoptimal regardless of HPV status. mSystems.

[19] Vargas-Robles D., Morales N., Rodríguez I., Nieves T., Godoy-Vitorino F., Alcaraz L.D., Pérez M.-E., Ravel J., Forney L.J., Domínguez-Bello M.G. (2020). Changes in the vaginal microbiota across a gradient of urbanization. Sci. Rep..

[20] Godoy-Vitorino F., Romaguera J., Zhao C., Vargas-Robles D., Ortiz-Morales G., Vázquez-Sánchez F., Sanchez-Vázquez M., de la Garza-Casillas M., Martinez-Ferrer M., White J.R. (2018). Cervicovaginal Fungi and Bacteria Associated With Cervical Intraepithelial Neoplasia and High-Risk Human Papillomavirus Infections in a Hispanic Population. Front. Microbiol..

[21] Godoy-Vitorino F., Romaguera J., Morales G.O., Vázquez-Sánchez F., Dominicci-Maura A., Ortiz A. (2019). Malassezia globosa and Malassezia restricta are associated with high-risk HPV infections in the anogenital tract. J. Low. Genit. Tract Dis..

[22] Godoy-Vitorino F., Ortiz-Morales G., Romaguera J., Sanchez M.M., Martinez-Ferrer M., Chorna N., Natarajaseenivasan K. (2018). Discriminating high-risk cervical Human Papilloma Virus infections with urinary biomarkers via non-targeted GC-MS-based metabolomics. PLoS ONE.

[23] Diaz-Rivera J., Rodríguez-Rivera M.A., Meléndez-Vázquez N.M., Godoy-Vitorino F., Dorta-Estremera S.M. (2024). Immune and Microbial Signatures Associated with PD-1 Blockade Sensitivity in a Preclinical Model for HPV+ Oropharyngeal Cancer. Cancers.

[24] Ruiz-Barrionuevo J.M., Kardas E., Rodríguez-Barreras R., Quiñones-Otero M.A., Ruiz-Diaz C.P., Toledo-Hernández C., Godoy-Vitorino F. (2024). Shifts in the gut microbiota of sea urchin Diadema antillarum associated with the 2022 disease outbreak. Front. Microbiol..

[25] Rodriguez-Barreras R., Tosado-Rodríguez E.L., Dominicci-Maura A., Godoy-Vitorino F. (2024). Effects of temperature and size class on the gut digesta microbiota of the sea urchin Tripneustes ventricosus. PeerJ.

[26] Rodriguez-Barreras R., Dominicci-Maura A., Tosado-Rodríguez E.L., Godoy-Vitorino F. (2023). The Epibiotic Microbiota of Wild Caribbean Sea Urchin Spines Is Species Specific. Microorganisms.

[27] Rodriguez-Barreras R., Tosado-Rodriguez E.L., Godoy-Vitorino F. (2021). Trophic niches reflect compositional differences in microbiota among Caribbean sea urchins. PeerJ.

[28] Lee S., Zhang A., Florets M.A., de Ángel Solá D., Cao L., Bolanos-Rosero B., Wang L., Godoy-Vitorino F., Matos N.R., Wang L. (2022). Prenatal exposure to Hurricane Maria is associated with an altered infant nasal microbiome. J. Allergy Clin. Immunol. Glob..

[29] Wang R., de Ángel Solá D., Rivera-Mariani F.E., Rosero B.B., Matos N.R., Wang L. (2025). Dysbiosis in the Nasal Mycobiome of Infants Born in the Aftermath of Hurricane Maria. Microorganisms.

[30] Ortiz A.P., Acosta-Pagán K.T., Oramas-Sepúlveda C., Castañeda-Avila M.A., Vilanova-Cuevas B., Ramos-Cartagena J.M., Vivaldi J.A., Pérez-Santiago J., Pérez C.M., Godoy-Vitorino F. (2022). Oral microbiota and periodontitis severity among Hispanic adults. Front. Cell. Infect. Microbiol..

[31] Sepúlveda-Rivera V., Olivieri-Henry G., Morales-González H., Ruiz-Adames J., Herrero-Rivera C., Rentas-Echeverria A., Cardona-Berdecia V., Soler-Llompart C., Sala-Morales A.C., Pérez-Montero G. (2025). Gut microbiota distinguishes aging hispanics with Alzheimer’s disease: Associations with cognitive impairment and severity. Sci Rep..

[32] Acosta-Pagán K., Bolaños-Rosero B., Pérez C., Ortíz A.P., Godoy-Vitorino F. (2024). Ecological Competition in the Oral Mycobiome of Hispanic adults Living in Puerto Rico associates with Periodontitis. J. Oral Microbiol..

[33] Stulberg E., Bolaños-Rosero B., Pérez C., Ortíz A.P., Godoy-Vitorino F. (2016). An assessment of US microbiome research. Nat. Microbiol..

[34] Martiny J.B.H., Whiteson K.L., Bohannan B.J.M., David L.A., Hynson N.A., McFall-Ngai M., Rawls J.F., Schmidt T.M., Abdo Z., Blaser M.J. (2020). The emergence of microbiome centres. Nat. Microbiol..

[35] Ruiz Barrionuevo J.M., Vilanova-Cuevas B., Alvarez A., Martín E., Malizia A., Galindo-Cardona A., de Cristóbal R.E., Occhionero M.A., Chalup A., Monmany-Garzia A.C. (2022). The Bacterial Fungal Gut Microbiota of the Greater Wax Moth, *Galleria mellonella* L. Consuming Polyethylene and Polystyrene. Front. Microbiol..

[36] Abarca J.G., Zuniga I., Ortiz-Morales G., Lugo A., Viquez-Cervilla M., Rodriguez-Hernandez N., Vázquez-Sánchez F., Murillo-Cruz C., Torres-Rivera E.A., Pinto-Tomás A.A. (2017). Characterization of the Skin Microbiota of the Cane Toad Rhinella cf. marina in Puerto Rico and Costa Rica. Front. Microbiol..

[37] Rook O., Zwart H. (2025). Awareness of human microbiome may promote healthier lifestyle and more positive environmental attitudes. Commun. Med..

